# Development and validation of an interpretable clinical scoring model to monitor the progression of preclinical Alzheimer’s disease

**DOI:** 10.1186/s13195-025-01931-3

**Published:** 2025-12-19

**Authors:** Chenyin Chu, Yihan Wang, Liwei Ma, Youjian Ouyang, Georgios Zisis, Colin L. Masters, Benjamin Goudey, Liang Jin, Yijun Pan, A4 and LEARN Study Teams

**Affiliations:** 1 School of Translational Medicine, Monash University, Melbourne, VIC 3004 Australia; 2 Florey Department of Neuroscience and Mental Health, The University of Melbourne, Parkville, VIC 3052 Australia; 3 Florey Institute of Neuroscience and Mental Health, Parkville, VIC 3052 Australia; 4 Department of Cardiology, Northern Hospital, Epping, VIC 3076 Australia; 5 Australian Biocommons, The University of Melbourne, North Melbourne, VIC 3051 Australia; 6 School of Health and Biomedical Sciences, Royal Melbourne Institute of Technology University, Bundoora,, VIC 3082 Australia

**Keywords:** Amyloid-beta, AutoScore, Machine learning, Phosphorylated tau-217, Plasma biomarkers, Preclinical Alzheimer’s disease, Prognostic modeling

## Abstract

**Background:**

Monitoring the progression of preclinical Alzheimer's disease (AD) is challenging due to the absence of obvious cognitive impairment, yet it's crucial for determining the optimal timing for disease-modifying treatments.

**Methods:**

This prognostic study used data from the Anti-Amyloid Treatment in Asymptomatic Alzheimer’s Disease (A4) study. The development cohort included 412 participants from the placebo arm, and external validation was performed using 342 participants from the treatment arm. All participants had baseline brain amyloid-beta (Aβ) positron emission tomography (PET) standardized uptake value ratios (SUVR) below 1.4. Using the AutoScore machine learning framework, we developed two interpretable models: the AutoScore Amyloid-Beta (ASAB) model to predict Aβ accumulation (> 1.4 SUVR) and the AutoScore Phosphorylated Tau (ASPT) model to predict plasma phosphorylated tau-217 (pTau-217) elevation (> 0.3 pg/mL) over 4.5 years. Model performance was assessed using the area under the receiver operating characteristic curve (AUC-ROC) with 95% confidence intervals (CIs).

**Results:**

The ASAB model achieved an AUC-ROC of 0.87 (95% CI, 0.79–0.95), and the ASPT model achieved 0.86 (95% CI, 0.78–0.94). Modified models excluding baseline Aβ SUVR or pTau-217 values maintained a strong performance (AUC-ROC 0.76–0.82). External validation demonstrated a robust performance, with an AUC-ROC of 0.83 (95% CI, 0.78–0.87) for ASAB and 0.84 (95% CI, 0.80–0.89) for ASPT. Key predictors included baseline biomarker levels, cholesterol, platelet count, alanine transaminase, and creatine kinase.

**Conclusions:**

The AutoScore-based models accurately predict longitudinal Aβ and pTau-217 levels using readily available clinical and laboratory data. These interpretable tools could help clinicians and researchers monitor preclinical AD progression and identify optimal windows for intervention.

**Supplementary Information:**

The online version contains supplementary material available at 10.1186/s13195-025-01931-3.

## Background

Recent research has significantly advanced our understanding of the blood-based biomarkers for Alzheimer’s disease (AD) [1–4]. Individuals with preclinical AD have underlying disease pathology, such as accumulation of amyloid-beta (Aβ) and hyperphosphorylated tau (pTau) in the brain, yet do not exhibit the cognitive symptoms typically associated with the disease [5–7], making it difficult to be detected. Recently, brain Aβ positron emission tomography (PET) imaging and plasma pTau-217 levels have been used to identify preclinical AD [8, 9]. These methods enable clinicians and researchers to recruit participants for trials focused on testing novel therapeutics and preventive interventions. It is widely believed that addressing the disease in its earliest stages may slow cognitive decline and ultimately improve patients’ quality of life [10, 11]. Although brain Aβ PET imaging is effective for monitoring progression, its high cost, limited availability, and resource requirements restrict its use in routine follow-up. Plasma pTau-217 offers a more scalable and cost-efficient alternative, although it is not yet widely available.

In this study, we aimed to develop prognostic models that predict Aβ and plasma pTau-217 positivity over a 4.5-year period. The models incorporate baseline Aβ PET SUVR or plasma pTau-217 levels, routine blood tests, clinical assessments, demographic characteristics, and genetic information. Model development was leveraged on the AutoScore framework, an interpretable machine learning approach that generates point-based clinical scoring systems [12]. AutoScore integrates feature ranking, optimal cut-off selection, score derivation, and parsimony analysis to produce simplified risk scores that can be readily applied in clinical settings without complex computation. The framework has been validated across multiple healthcare applications and is designed to balance predictive accuracy with interpretability [13–16], making it well suited for identifying individuals with preclinical AD that may have higher amyloid burden over 4.5 years. After feature selection through AutoScore, the selected features were further examined using epidemiological analyses. Model development was performed using participants from the placebo arm of the Anti-Amyloid Treatment in Asymptomatic Alzheimer’s Disease (A4) trial, and external validation was conducted in the treatment arm to assess generalizability.

## Methods

### Data sources and ethics

This study utilized de-identified data from the A4 study [17, 18]. The A4 Study design has been described previously and briefly summarized in the Supplementary Materials. The full study protocol is publicly available at a4studydata.org. The A4 Study received approval from institutional review boards at all participating sites in the United States, Canada, Australia, and Japan. Data access was obtained through the official A4 data portal. Institutional ethics approval to analyze de-identified human data was granted by Monash University (Project ID 48297).

The A4 Study is a phase 3 secondary prevention trial enrolling cognitively unimpaired older adults with elevated amyloid burden on florbetapir PET. Participants were randomized to receive solanezumab or placebo and were followed for 240 weeks with repeated amyloid PET and plasma biomarker assessments. Amyloid PET imaging was performed using the 18F-florbetapir (AV-45) tracer, and standardized uptake value ratios (SUVRs) were calculated [17, 18]. PET acquisition and quantification followed the harmonized procedures of the A4 protocol. Plasma pTau-217 concentrations were measured using the Eli Lilly–developed immunoassay on the Meso Scale Discovery platform in the A4 study [19, 20].

### Study design and participants

This prognostic study used machine learning models to predict whether brain Aβ or plasma pTau-217 levels would exceed predefined thresholds over 4.5 years. Model development followed the TRIPOD + AI guideline for prognostic machine learning (ML) research [21]. Biomarker thresholds were defined based on prior literature: SUVR > 1.4 for elevated brain amyloid burden [22–24] and plasma pTau-217 > 0.3 pg/mL for abnormal levels [25–27]. All candidate predictors included in this study are listed in eTable 1 and categorized into demographic, genetic, clinical, laboratory, plasma biomarker, PET imaging, and cognitive domains.

### Inclusion and exclusion criteria

Participants who were randomized to either placebo or solanezumab and who had amyloid PET SUVR and plasma pTau-217 measurements available at both baseline and 240 weeks were eligible. Of the initial 1,147 enrolled individuals (583 in the placebo arm and 564 in the solanezumab arm), 412 placebo participants were included for model development and internal validation. For external validation, 342 participants randomized to solanezumab were analyzed.

### Model development and evaluation

We used the AutoScore framework, an interpretable ML tool that automates the construction of point-based clinical scoring models for predicting brain amyloid and plasma pTau-217 burden [12, 28]. The AutoScore pipeline consists of five modules: 1) feature ranking, 2) feature transformation, 3) score derivation, 4) model selection, and 5) performance evaluation. A visual overview of the modeling workflow is provided in eFigure 1, with technical details in eMethods. The dataset was split into a training set (70%) and a test set (30%). The training set was used for modules 1–4, and the test set was used for module 5.

Two primary models were developed: the AutoScore Amyloid-Beta (ASAB) model for predicting elevated brain Aβ and the AutoScore phosphorylated tau (ASPT) model for predicting elevated plasma pTau-217. Both models incorporated all available predictors (eTable 1). Modified versions of each model were also created after intentionally excluding baseline Aβ SUVR or pTau-217 values to simulate real-world settings in which these assays, particularly plasma pTau-217 assay is not widely available. This allowed evaluation of the models’ applicability in resource-variable environments and assessment of the incremental predictive value contributed by pTau-217 when available. All full and modified models were developed using identical procedures to ensure direct comparability.

Model performance was evaluated using the area under the receiver operating characteristic curve (AUC-ROC), sensitivity, specificity, positive predictive value (PPV), and negative predictive value (NPV). Bias-corrected 95% confidence intervals (CIs) for all metrics were estimated using 100 bootstrap resamples. The ASAB and ASPT models were further compared with two baseline models: logistic regression with random forest-based feature selection (“logistic-select”) and logistic regression including all candidate predictors (“logistic-all”). Generalizability was assessed by applying ASAB and ASPT to the 342 participants in the treatment arm. To support potential clinical use, we developed a lightweight, web-based ASAB/ASPT risk calculator that allows clinicians to enter available predictors and receive individualized 4.5-year estimates of future Aβ or pTau-217 burden. The tool is freely accessible at: https://jackchu.shinyapps.io/shiny/.

### Epidemiology study

Epidemiological analyses were conducted to complement the ML findings. All participants in the placebo arm (*n* = 412) were included. Multivariable linear regression models were used to examine associations between baseline characteristics and Aβ accumulation or plasma pTau-217 levels at 4.5 years, adjusting for age, sex, education, and apolipoprotein E (APOE) ε4 carrier status. Results are reported as beta (β) coefficients with 95% CIs. Continuous variables were standardized to z-scores (mean = 0, SD = 1).

### Statistical analysis

Data preprocessing and analysis were conducted using Python 3.9 with the *pandas*, *NumPy*, and *scikit-learn* libraries. Additional analyses were performed in RStudio (version 2023.12.0 + 369) using the *tidyverse* and *caret* packages, and in Stata version 17.0. Predictive modeling used the AutoScore package in R 3.5.3, which automates derivation of point-based scoring systems [28]. Model performance metrics and 95% CIs were estimated using bootstrap resampling. Comparisons of AUC-ROC values between the AutoScore models and the logistic regression baselines were performed using the Wilcoxon signed-rank test (via the *rstatix* package [29]), which is appropriate for paired AUC estimates derived from bootstrap samples [30].

## Results

### Participant characteristics

Baseline characteristics stratified by week-240 biomarker outcomes are presented in Table 1. Across both ASAB and ASPT cohorts, the mean baseline age remained consistent at approximately 71–72 years, and females outnumbered males in all subgroups. Education levels were similar (approximately 16–17 years), and the proportion of APOE ε4 carriers exceeded that of non-carriers in both treatment and placebo arms.

**Table 1:** A4-Study participants characteristic

**Group**	ASAB (placebo)* n* = 289		ASAB (treatment)* n* = 342		ASPT (placebo) * n* = 292		ASPT (treatment)* n* = 289	
	240-week Aβ SUVR ≥ 1.4 *n* = 187	240-week Aβ SUVR < 1.4*n* = 102	240-week Aβ SUVR ≥ 1.4 *n* = 174	240-week Aβ SUVR < 1.4*n* = 168	240-week pTau-217 ≥ 0.3 pg/ml *n* = 172	240-week pTau-217 < 0.3 pg/ml *n* = 120	240-week pTau-217 ≥ 0.3 pg/ml *n* = 127	240-week pTau-217 < 0.3 pg/ml *n* = 162
Age at baseline (year)	71.70 (4.64)	71.86 (4.59)	72.00 (4.46)	71.23 (4.40)	72.24 (4.66)	71.05 (4.38)	72.46 (4.27)	70.73 (4.19)
Sex	Female/Male = 115/72	Female/Male = 54/48	Female/Male = 107/67	Female/Male = 93/75	Female/Male = 110/62	Female/Male = 62/58	Female/Male = 79/48	Female/Male = 92/70
Education (year)	16.40 (2.89)	16.72 (2.69)	16.26 (2.47)	16.82 (2.45)	16.47 (2.77)	16.57 (2.86)	16.16 (2.49)	16.93 (2.46)
APOE ε4 carrier	Yes/No = 139/48	Yes/No = 56/47	Yes/No = 124/50	Yes/No = 99/69	Yes/No = 118/54	Yes/No = 78/42	Female/Male = 80/47	Female/Male = 110/52
PACC	0.05 (2.66)	0.35 (2.51)	−0.09 (2.72)	0.37 (2.59)	0.02 (2.71)	0.40 (2.45)	−0.54 (2.85)	0.74 (2.39)
pTau-217 (pg/mL)								
Baseline	0.30 (0.12)	0.23 (0.10)	0.34 (0.17)	0.25 (0.13)	0.33 (0.12)	0.20 (0.06)	0.34 (0.15)	0.21 (0.06)
Week 240	n/a^~^		n/a^~^		0.49 (0.19)	0.23 (0.04)	0.48 (0.23)	0.23 (0.04)
Brain amyloid burden (SUVR)								
Baseline	1.37 (0.15)	1.20 (0.08)	1.38 (0.16)	1.24 (0.12)	1.35 (0.16)	1.26 (0.15)	1.41 (0.18)	1.27 (0.14)
Week 240	1.52 (0.14)	1.27 (0.10)	1.53 (0.13)	1.28 (0.09)	n/a^#^		n/a^#^	

1) 169 participants were shared between the ASAB and ASPT placebo groups, while 284 participants were shared between the ASAB and ASPT treatment groups

2) ~ not all ASAB participants have plasma pTau-217 levels recorded; ^#^not all ASPT participants have brain amyloid SUVR recorded

In the ASAB groups, baseline brain amyloid SUVR values were comparable between individuals who later exceeded the SUVR ≥ 1.4 threshold and those who did not, although week-240 SUVR values were higher among participants who crossed the threshold. In the ASPT groups, baseline pTau-217 concentrations were similar across corresponding subgroups, with week-240 values higher among individuals who exceeded the ≥ 0.3 pg/mL threshold.

### ASAB model development and evaluation

**Fig. 1:**
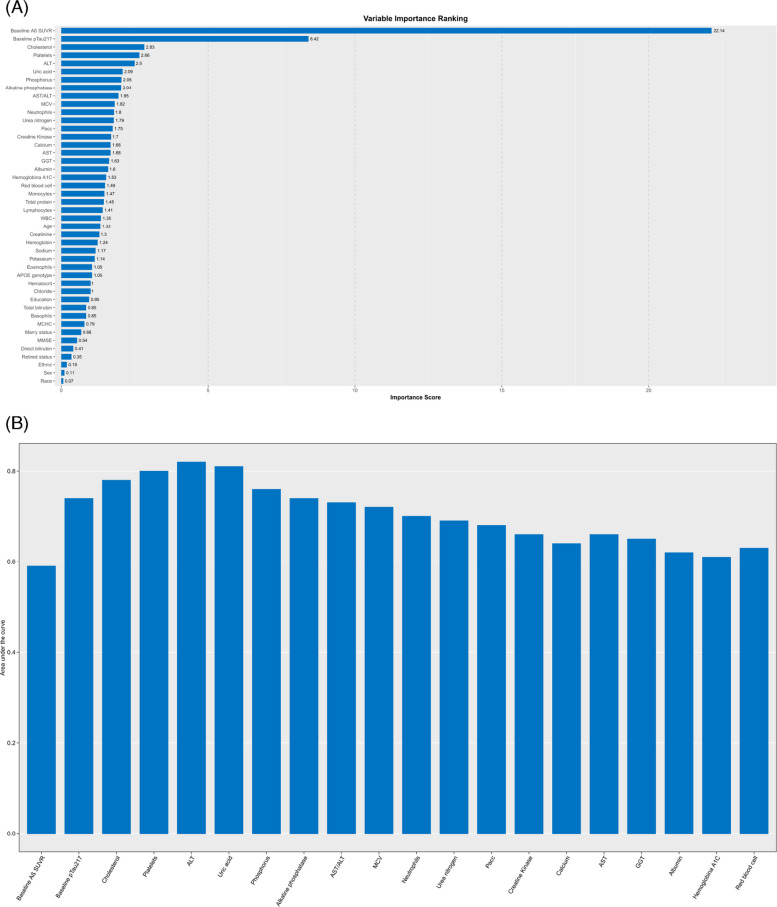
Auto-S-score amyloid-beta model. **A** The feature importance ranking plot identifies the most influential features associated with the 4.5-year future brain amyloid burden. **B** The parsimony plot illustrates the ASAB model’s performance on the training set as a function of the number of features included

Feature importance ranking for the ASAB model is shown in Fig. 1A. Baseline brain Aβ was the strongest predictor, followed by baseline plasma pTau-217 and several routine blood results, including cholesterol, platelet count, alanine transaminase (ALT), uric acid, and phosphorus. As illustrated in the parsimony plot (Fig. 1B), the AUC-ROC increased from 0.62 using baseline brain Aβ alone to 0.79 with the addition of baseline pTau-217 and peaked at 0.83 with the top five features. Adding more than five features did not improve performance but instead reduced the AUC-ROC; therefore, the top five predictors were selected for the ASAB model. Scores for each selected feature category were derived using multivariable logistic regression, with the total score scaled to a maximum of 100 (Table 2). An optimal cutoff of 45 was identified for predicting high Aβ burden. The ASAB model demonstrated a strong performance, achieving an AUC-ROC of 0.87 (95% CI, 0.79–0.95), sensitivity of 0.76 (95% CI, 0.61–0.91), specificity of 0.86 (95% CI, 0.79–0.94), PPV of 0.93 (95% CI, 0.85–0.97), and NPV of 0.86 (95% CI, 0.76–0.96).

**Table 2:** Score tables

Features of ASAB model	Interval	Score
baseline brain amyloid (SUVR)	< 1.21	0
	[1.21, 1.33)	21
	[1.33, 1.45)	39
	≥ 1.45	56
baseline pTau-217 (pg/mL)	< 0.19	3
	[0.19, 0.24)	10
	[0.24, 0.32)	4
	≥ 0.32	0
blood test-platelets (10^9^/L)	< 184	3
	[184, 223)	0
	[223, 262)	6
	≥ 262	16
blood test-ALT (U/L)	< 14	5
	[14, 17)	8
	[17, 21)	1
	≥ 21	0
blood test- cholesterol (mmol/L)	< 4.28	10
	[4.28, 4.96)	0
	[4.96, 5.66)	6
	≥ 5.66	7

Features of ASPT model	Interval	Score
baseline pTau-217 (pg/mL)	< 0.186	0
	[0.186, 0.244)	20
	[0.244, 0.323)	28
	≥ 0.323	46
baseline brain amyloid (SUVR)	< 1.2	0
	[1.2, 1.33)	7
	[1.33, 1.45)	10
	≥ 1.45	12
blood test—creatine kinase (U/L)	< 69	8
	[69, 92)	8
	[92, 132)	10
	≥ 132	0
electrocardiogram—R–R interval (millisecond)	< 853	0
	[853, 954)	2
	[954, 1050)	6
	≥ 1050	8
blood test – cholesterol (mmol/L)	< 4.37	4
	[4.37, 5.04)	0
	[5.04, 5.81)	7
	≥ 5.81	10
blood test – eosinophils (10^9^/L)	< 0.09	0
	[0.09, 0.13)	10
	[0.13, 0.20)	12
	≥ 0.20	5

Given that baseline Aβ SUVR is not always available in clinical practice, we developed a modified ASAB model excluding this variable. The model achieved an AUC-ROC of 0.80 (95% CI, 0.71–0.90), sensitivity of 0.70 (95% CI, 0.67–0.74), specificity of 0.78 (95% CI, 0.76–0.86), PPV of 0.89 (95% CI, 0.80–0.94), and NPV of 0.77 (95% CI, 0.70–0.87). A second modified ASAB model excluding baseline pTau-217 achieved an AUC-ROC of 0.82 (95% CI, 0.77–0.88), with sensitivity of 0.79 (95% CI, 0.70–0.86), specificity of 0.78 (95% CI, 0.75–0.82), PPV of 0.82 (95% CI, 0.76–0.88), and NPV of 0.73 (95% CI, 0.71–0.76). Feature rankings, parsimony plots, and score tables for the modified models are shown in eFigures 2, 3 and eTable 2. Collectively, these findings highlight the strong potential of the ASAB models to predict whether brain amyloid burden exceeds the SUVR threshold of 1.4 in preclinical AD.

### ASPT model development and evaluation

**Fig. 2:**
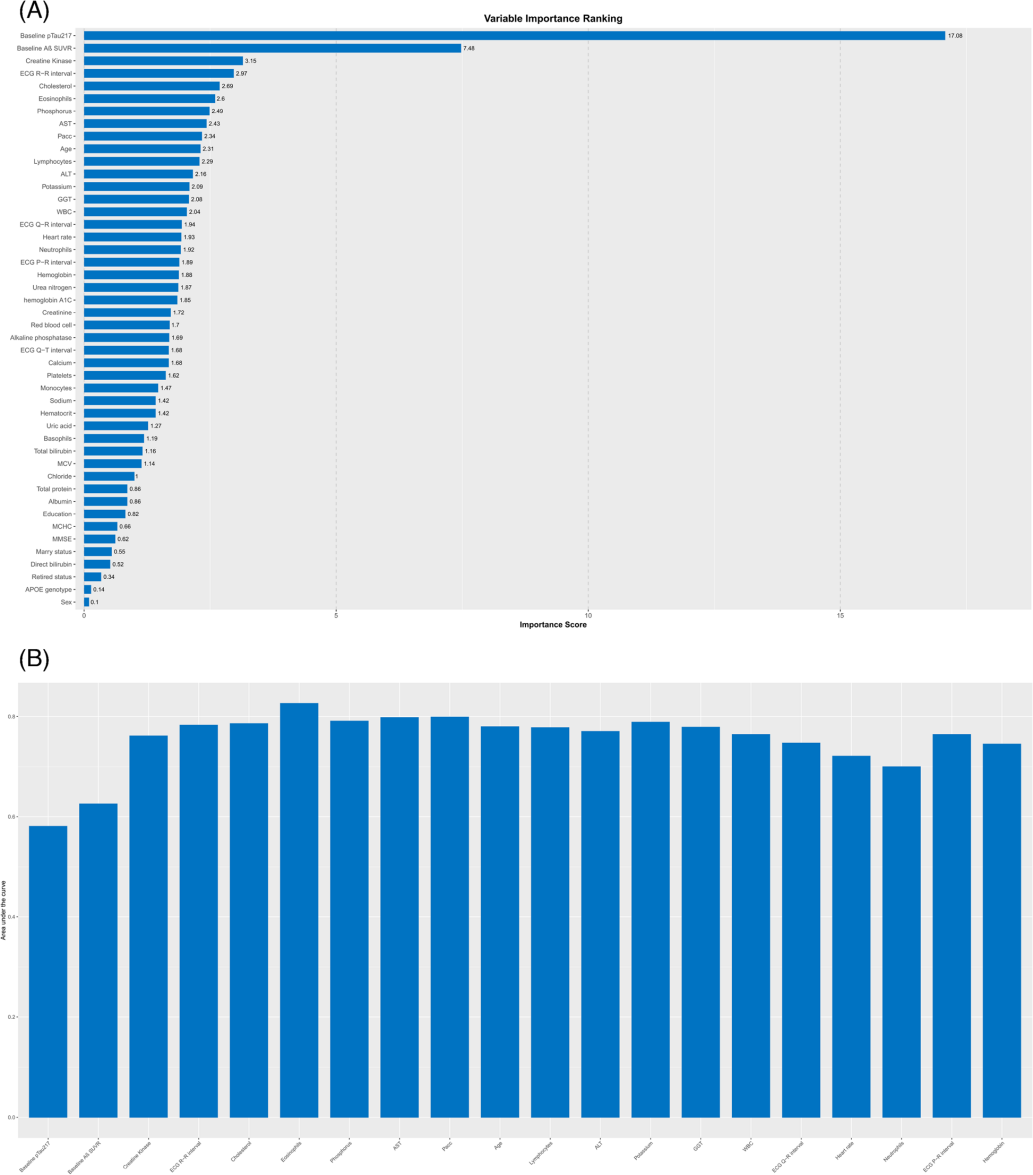
AutoScore plasma pTau-217 model. **A** The feature importance of the plot highlights the most influential features associated with the 4.5-year future plasma pTau-217 levels. **B** The parsimony plot illustrates the performance of the ASPT model on the training set as a function of the number of features included

Feature importance ranking for the ASPT model is shown in Fig. 2A. Baseline pTau-217 was the strongest predictor, followed by baseline brain Aβ, creatine kinase, cholesterol, ECG R-R interval, and eosinophil count. Parsimony analysis (Fig. 2B) showed a gradual improvement in AUC-ROC from 0.58 to 0.83 as features were added up to the sixth feature. Beyond six features, no further performance gain was observed, indicating that six predictors were optimal. The final scoring table (Table 2) was scaled to a maximum of 100. Using a threshold score of 54, the ASPT model achieved an AUC-ROC of 0.86 (95% CI, 0.78–0.94), with sensitivity of 0.87 (95% CI, 0.77–0.94), specificity of 0.71 (95% CI, 0.67–0.86), PPV of 0.82 (95% CI, 0.74–0.90), and NPV of 0.78 (95% CI, 0.67–0.90).

A modified ASPT model excluding baseline Aβ burden achieved an AUC-ROC of 0.81 (95% CI, 0.72–0.91), with sensitivity of 0.86 (95% CI, 0.76–0.94), specificity of 0.72 (95% CI, 0.68–0.86), PPV of 0.81 (95% CI, 0.74–0.90), and NPV of 0.79 (95% CI, 0.68–0.90). A second modified ASPT model excluding baseline pTau-217 achieved an AUC-ROC of 0.76 (95% CI, 0.70–0.86), with sensitivity of 0.71 (95% CI, 0.65–0.74), specificity of 0.83 (95% CI, 0.70–0.88), PPV of 0.84 (95% CI, 0.78–0.86), and NPV of 0.70 (95% CI, 0.62–0.80). Supporting illustrations and score tables are provided in eFigures 4, 5 and eTable 3. Overall, these results demonstrate that the ASPT models effectively predict plasma pTau-217 positivity in preclinical AD.

### Comparison between the baseline models and ASAB/ASPT models

Comparisons between the AutoScore models and baseline logistic regression models are summarized in Table 3. The ASAB model outperformed the logistic-all model, which achieved an AUC-ROC of 0.73 (95% CI, 0.61–0.78), sensitivity of 0.71 (95% CI, 0.56–0.81), specificity of 0.70 (95% CI, 0.52–0.83), PPV of 0.81 (95% CI, 0.72–0.86), and NPV of 0.57 (95% CI, 0.45–0.68). The logistic-select model performed better (AUC-ROC 0.80; 95% CI, 0.74–0.81) but still underperformed compared with ASAB model.

**Table 3:** Comparison between ASAB/ASPT models and baseline models

Model	AUC-ROC	Sensitivity	Specificity	PPV	NPV
Amyloid beta prediction					
ASAB model	0.87 (95%CI: 0.79–0.95)	0.76 (95%CI: 0.61–0.91)	0.86 (95%CI: 0.79–0.94)	0.93 (95%CI: 0.85–0.97)	0.86 (95%CI: 0.76–0.96)
Logistic-all	0.73 (95%CI: 0.61–0.78) ***P* < 0.001**	0.71 (95%CI: 0.56–0.81)	0.70 (95%CI: 0.52–0.83)	0.81 (95%CI: 0.72–0.86)	0.57 (95%CI: 0.45–0.68)
Logistic-select	0.80 (95%CI: 0.74–0.81) ***P* < 0.001**	0.72 (95%CI: 0.67–0.78)	0.76 (95%CI: 0.60–0.80)	0.82 (95%CI: 0.81–0.83)	0.67 (95%CI: 0.58–0.71)
Amyloid beta prediction, without baseline bran amyloid SUVR					
ASAB model	0.80 (95%CI: 0.71–0.90)	0.70 (95%CI: 0.67–0.74)	0.78 (95%CI: 0.76–0.86)	0.89 (95%CI: 0.80–0.94)	0.77 (95%CI: 0.70–0.87)
Logistic-all	0.64 (95%CI: 0.55–0.70) ***P* < 0.001**	0.75 (95%CI: 0.65–0.82)	0.57 (95%CI: 0.37–0.63)	0.76 (95%CI: 0.64–0.78)	0.55 (95%CI: 0.34–0.59)
Logistic-select	0.68 (95%CI: 0.63–0.76) ***P* < 0.001**	0.81 (95%CI: 0.72–0.88)	0.53 (95%CI: 0.46–0.54)	0.68 (95%CI: 0.64–0.77)	0.58 (95%CI: 0.50–0.61)
Amyloid beta prediction, without baseline plasma pTau-217 level					
ASAB model	0.82 (95%CI: 0.77–0.88)	0.79 (95%CI: 0.70–0.86)	0.78 (95%CI: 0.75–0.82)	0.82 (95%CI: 0.76–0.88)	0.73 (95%CI: 0.71–0.76)
Logistic-all	0.70 (95%CI: 0.62–0.75) ***P* < 0.001**	0.66 (95%CI: 0.53–0.75)	0.68 (95%CI: 0.53–0.78)	0.73 (95%CI: 0.63–0.79)	0.60 (95%CI: 0.55–0.68)
Logistic-select	0.73 (95%CI: 0.69–0.74) ***P* < 0.001**	0.72 (95%CI: 0.68–0.80)	0.75 (95%CI: 0.65–0.83)	0.77 (95%CI: 0.68–0.80)	0.72 (95%CI: 0.63–0.77)
pTau-217 prediction					
ASPT model	0.86 (95%CI: 0.78–0.94)	0.87 (95%CI: 0.77–0.94)	0.71 (95%CI: 0.67–0.86)	0.82 (95%CI: 0.74–0.90)	0.78 (95%CI: 0.67–0.90)
Logistic-all	0.71 (95%CI: 0.58–0.73) ***P* < 0.001**	0.72 (95%CI: 0.59–0.79)	0.56 (95%CI: 0.43–0.63)	0.70 (95%CI: 0.63–0.78)	0.59 (95%CI: 0.47–0.66)
Logistic-select	0.76 (95%CI: 0.73–0.78) ***P* < 0.001**	0.72 (95%CI: 0.63–0.80)	0.70 (95%CI: 0.54–0.76)	0.77 (95%CI: 0.70–0.80)	0.64 (95%CI: 0.56–0.72)
pTau-217 prediction, without baseline brain amyloid SUVR					
ASPT model	0.81 (95%CI: 0.72–0.91)	0.86 (95%CI: 0.76–0.94)	0.72 (95%CI: 0.68–0.86)	0.81 (95%CI: 0.74–0.90)	0.79 (95%CI: 0.68–0.90)
Logistic-all	0.67 (95%CI: 0.59–0.70) ***P* < 0.001**	0.64 (95%CI: 0.50–0.66)	0.71 (95%CI: 0.52–0.77)	0.72 (95%CI: 0.63–0.77)	0.62 (95%CI: 0.47–0.67)
Logistic-select	0.74 (95%CI: 0.72–0.75) ***P* < 0.001**	0.70 (95%CI: 0.68–0.77)	0.68 (95%CI: 0.66–0.74)	0.74 (95%CI: 0.68–0.78)	0.74 (95%CI: 0.65–0.80)
pTau-217 prediction, without baseline plasma pTau-217 level					
ASPT model	0.76 (95%CI: 0.70–0.86)	0.71 (95%CI: 0.65–0.74)	0.83 (95%CI: 0.70–0.88)	0.84 (95%CI: 0.78–0.86)	0.70 (95%CI: 0.62–0.80)
Logistic-all	0.60 (95%CI: 0.51–0.65) ***P* < 0.001**	0.63 (95%CI: 0.55–0.65)	0.58 (95%CI: 0.43–0.63)	0.68 (95%CI: 0.62–0.71)	0.52 (95%CI: 0.43–0.63)
Logistic-select	0.67 (95%CI: 0.60–0.69) ***P* < 0.001**	0.66 (95%CI: 0.58–0.74)	0.67 (95%CI: 0.52–0.73)	0.64 (95%CI: 0.60–0.68)	0.59 (95%CI: 0.53–0.64)

The *P*-value is derived from the Wilcoxon signed-rank test, comparing the AUC-ROC values of ASAB/ASPT against logistic-all and ASAB/ASPT against logistic-select.

Similarly, the ASPT model demonstrated a superior performance relative to the logistic-all and logistic-select models, which achieved AUC-ROC values of 0.70 (95% CI, 0.62–0.75) and 0.73 (95% CI, 0.69–0.74), respectively. Modified ASAB and ASPT models also outperformed their respective baseline models (Table 3). Together, these findings highlight the robustness and predictive strength of the AutoScore models.

### External evaluation results

The ASAB model was externally evaluated using 342 participants from the A4 treatment arm. Using a threshold score of 45 and the corresponding scoring table (Table 2), the model achieved an AUC-ROC of 0.83 (95% CI, 0.78–0.87), sensitivity of 0.78 (95% CI, 0.72–0.84), specificity of 0.77 (95% CI, 0.70–0.83), PPV of 0.78 (95% CI, 0.73–0.83), and NPV of 0.77 (95% CI, 0.72–0.82). External validation of the ASPT model included 289 treatment-arm participants. With a threshold score of 54, the model achieved an AUC-ROC of 0.84 (95% CI, 0.80–0.89), sensitivity of 0.77 (95% CI, 0.70–0.85), specificity of 0.80 (95% CI, 0.74–0.86), PPV of 0.76 (95% CI, 0.70–0.82), and NPV of 0.82 (95% CI, 0.77–0.87).

### Epidemiology analysis

Epidemiological analyses were performed to corroborate the machine learning findings (eTable 4). After adjusting for age, sex, education, and APOE ε4 carrier status, baseline brain Aβ (β = 0.808, *P* < 0.001) and plasma pTau-217 (β = 0.331, *P* < 0.001) were positively associated with brain Aβ accumulation after 4.5 years. Higher baseline PACC scores were inversely associated with brain amyloid burden (β = –0.171, *P* < 0.001). Higher baseline ALT was associated with a lower brain Aβ accumulation at 4.5 years (β = –0.136, *P* = 0.006). For plasma pTau-217 outcomes, baseline brain Aβ (β = 0.432, *P* < 0.001) and baseline plasma pTau-217 (β = 0.837, *P* < 0.001) were positively associated with the Week-240 plasma pTau-217 levels. Higher baseline PACC scores were inversely associated (β = –0.191, *P* < 0.001). No significant associations were observed between routine blood test measures and plasma pTau-217 at follow-up.

## Discussion

In this study, we developed the ASAB and ASPT models to predict brain Aβ burden and plasma pTau-217 levels over 4.5 years in individuals with preclinical AD. For the ASAB models, the strongest predictors of subsequent Aβ burden were baseline amyloid PET SUVR, baseline pTau-217 levels (pg/mL), cholesterol levels (mmol/L), platelet count, and serum ALT (Fig. 1A, eFigure 2). According to our score tables, higher cholesterol levels within the normal range were associated with higher risk scores, suggesting that cholesterol may contribute to brain Aβ accumulation. Similar findings have been reported previously, with higher cholesterol levels associated with clinical manifestations of AD [31]. However, the relationship between cholesterol and AD risk has not been consistently observed across all studies [32]. Platelets are a major source of circulating Aβ precursor proteins, and upon activation, they can secrete substantial amounts of Aβ into the bloodstream. This Aβ can then be transported across the blood–brain barrier and contribute to amyloid deposition [33]. Serum ALT may serve as a metabolic indicator that indirectly reflects the liver’s capacity to process amyloid precursors. Our score table suggested that lower ALT levels may be associated with higher brain amyloid accumulation, consistent with a previous report [34].

For the ASPT models, creatine kinase and eosinophil counts were consistently identified as strong predictors of plasma pTau-217 levels. Our scoring system indicated that lower creatine kinase levels were associated with higher plasma pTau-217 levels. Notably, models that used either baseline brain Aβ or baseline pTau-217 alone achieved performance comparable to models that incorporated both predictors. This finding may be explained by the strong correlation between plasma pTau-217 and brain Aβ in preclinical AD [35].

Our epidemiological analysis supported the associations identified by the machine learning models. The consistency between these approaches highlights the robustness of the relationships between baseline AD biomarker levels (brain Aβ and pTau-217), PACC scores, and subsequent trajectories of these biomarkers over 4.5 years in preclinical AD. The observed protective association of higher ALT levels with lower brain Aβ burden also aligned with the machine learning findings. Incorporating routine blood tests and plasma pTau-217 improved the accuracy of predicting Aβ burden. Consistent with recent work, pTau-217 enhanced diagnostic performance and represents an important advancement in Aβ detection [36, 37]. Although most associations between routine blood tests and AD biomarkers in preclinical AD did not reach statistical significance, consistent trends were observed, supporting the value of these measures as candidates for further investigation. The mechanisms by which routine blood markers contribute to predictive performance remain unclear and warrant exploration in future studies.

The potential utility of the ASAB and ASPT models in both clinical and research settings merits careful consideration. In research contexts, the required input features are relatively easy to obtain, making these models practical tools for identifying and recruiting individuals with preclinical AD who exhibit biomarker progression patterns aligned with specific study objectives. In clinical practice, further evaluation will be needed to determine how these models can be effectively integrated into existing healthcare systems. Their greatest benefit may emerge when disease-modifying or preventive interventions for preclinical AD become widely available.

By applying advanced ML methods, we developed interpretable predictive models that are accessible and potentially scalable for clinical use. An important feature of this work is the incorporation of routine blood test parameters alongside plasma pTau-217, an emerging and highly specific biomarker of AD pathology [9, 38], thereby enhancing the ability to predict whether an individual’s brain amyloid burden will exceed a predefined threshold. The integration of ML with epidemiological analysis offers a robust framework for characterizing these relationships and provides a strong foundation for future research.

In recent years, the integration of interpretable artificial intelligence into clinical workflows has gained increasing attention, as transparency and usability are essential for translating predictive models into real-world practice [39, 40]. Interpretable ML approaches, such as AutoScore, enable clinicians to understand how individual predictors contribute to risk estimation, thereby enhancing trust, accountability, and adoption in clinical decision-making [41, 42]. Our web-based ASAB/ASPT calculator exemplifies this concept by providing an intuitive interface that delivers individualized risk estimates without requiring technical expertise. Because the models are implemented as simple point-based scores and a web-based calculator, users are not required to manage complex data preprocessing, and no expertise in machine learning is needed. Such tools represent a movement to practical, explainable artificial intelligence solutions for disease monitoring.

Several limitations should be acknowledged. The A4 Study dataset was derived from a clinical trial population, which may not fully represent the broader preclinical AD population and may limit the generalizability of our findings. Future studies should aim to validate these models in more diverse and representative cohorts. In addition, although external evaluation demonstrated the robustness of the ASAB and ASPT models, the validation sample was drawn from the same overarching trial as the development cohort. This partial dependence underscores the need for further validation using fully independent datasets to confirm the reliability and broader applicability of the proposed models.

## Conclusions

We developed and validated two interpretable machine learning models, ASAB and ASPT, to predict brain Aβ burden and plasma pTau-217 levels in preclinical AD. Both models demonstrated high accuracy and maintained strong performance even when baseline biomarker measurements were unavailable, supporting their robustness and clinical feasibility. By integrating routine laboratory and demographic features with plasma biomarkers, these tools offer accessible and scalable approaches for monitoring disease progression before the onset of cognitive symptoms. Their application could help identify individuals at highest risk and guide early preventive or therapeutic strategies. Further validation in independent and more diverse cohorts will be essential to establish their generalizability and clinical utility. This study also reveals intriguing associations between routine biochemical markers and AD biomarkers, which warrant further mechanistic investigation. The AD biomarker prediction models developed here may provide important insights for clinicians when designing patient care plans, particularly once disease-modifying treatments become available for use in preclinical AD.

## Supplementary Information

Supplementary Material 1.

Supplementary Material 2.

## Data Availability

C.C. and Y.P. had full access to all the data in the study and took responsibility for the integrity of the data and the accuracy of the data analysis. Data used in the preparation of this article were obtained from A4 study ([http://a4studydata.org] (http://a4studydata.org)). The code used in this study can be made available upon making written requests to the first author (C.C.) and corresponding author (Y.P.). A4 data can be requested by submitting a request on [http://a4studydata.org] (http://a4studydata.org).

## Abbreviation

AD: Alzheimer’s disease
ALT: Alanine transaminase
APOE: Apolipoprotein E
ASAB: AutoScore Amyloid-Beta model
ASPT: AutoScore Phosphorylated Tau model
AUC-ROC: Area under the receiver operating characteristic curve
Aβ: Amyloid-beta
CI: Confidence interval
NPV: Negative predictive value
PACC: Preclinical Alzheimer’s Cognitive Composite
PET: Positron emission tomography
PPV: Positive predictive value
pTau-217: Phosphorylated tau-217
SUVR: Standardized uptake value ratio
TRIPOD: Transparent reporting of a multivariable prediction model for individual prognosis or diagnosis
