# Supplementary material for: Development and validation of an interpretable clinical scoring model to monitor the progression of preclinical Alzheimer’s disease
Source: Alzheimers Res Ther. 2025 Dec 19;17:268. doi: 10.1186/s13195-025-01931-3 (PMC12750953; doi:10.1186/s13195-025-01931-3)
Supplement: Supplementary file 2 — Supplementary Material 2. [file 13195_2025_1931_MOESM2_ESM.docx]

**Supplementary Materials**

**Table of Contents**

**(A) eMethods**

Auto-score algorithm

Description of the A4-Study

**(B) eTable**

eTable 1: Initial features (available as a separate excel file)

eTable 2: Score table for modified ASAB models

eTable 3: Score table for modified ASPT models

eTable 4: Epidemiology analysis

**(C) eFigure**

eFigure 1: Flowchart of the model development

eFigure 2: Feature ranking for modified ASAB models

eFigure 3: Parsimony plots for modified ASAB models

eFigure 4: Feature ranking for modified ASPT models

eFigure 5: Parsimony plots for modified ASPT models

**eMethods - auto-score algorithm**

The modified auto-score algorithm consists of five modules. Data from 70% of the participants (training set) and 30% of the participants (test set) were used for modules 1-4 and module 5. Steps of data handling steps are provided here to ensure results reproducibility.

**Data handling**

Step 1: Obtain A4-Study data through a formal application ([a4studydata.org](https://www.a4studydata.org/)).

Step 2: Review the data and select participants with complete records of interest for model development. The data should be divided into a training set (70%) and a test set (30%).

Step 3: Use RStudio version 12.0+369 to run modules 1-4 of the modified auto-score algorithm to obtain the score table and the thresholds for brain amyloid SUVR and plasma pTau-217 positivity. Ensure that the *auto-score* package is installed in your R programming environment (<https://github.com/nliulab/AutoScore>).

Step 4: Use the FDRS score table and threshold for AD to evaluate the model on test set and/or external dataset.

*Remark:* A detailed data handling and auto-score algorithm, developed by the package creator, has been implemented and is fully operational. A step-by-step flowchart outlining the process can be found in STAR Protocols[1].

**Module 1: Feature ranking**

Random forest (RF), an ensemble machine learning algorithm, has been used to identify the highest-ranking features for generating the score. RF comprises multiple tree-structured classifiers known as decision trees. Each tree is developed using a classification or regression tree method, and the final output is derived from the collective results of all trees, enhancing RF's robustness against overfitting[2]. For classification tasks, the Gini index is employed to determine the optimal split. Minimizing the Gini index reduces the probability of misclassification[3]. One advantage of using RF over other methods, such as backward stepwise regression or LASSO, is its ability to effectively rank features based on their nonlinear and heterogeneous impacts[4]. In the auto-score framework, the final list of features is determined by their importance rankings, alongside the parameter *m*, which represents the number of features ultimately selected. The value of *m* can be chosen on a case-by-case basis to align with clinical preferences, expert or domain knowledge, or the requirements of real-world applications.

**Module 2: Variable transformation**

All selected features undergo pre-processing and transformation. Specifically, continuous variables are converted into categorical variables to facilitate the modeling of nonlinear effects. This is a common approach to handling medical data to minimize the effects of outliers[5]. In the auto-score framework, the maximum number of categories for each variable is predefined to ensure practicality in the final risk score. For example, if the maximum number of categories is set at five, a continuous variable will be segmented into four intervals at the 0%, $25$%, $50$%, $75$%, and 100% scores of the variable's total value.

**Module 3: Score derivation**

Selected and transformed features are utilized to construct a risk score for predicting outcomes. Each category within these features is assigned a weight and given an integer point value. Multivariable logistic regression is employed to determine the weights for these scores. The formula is provided below:

$$P\left( Y=1 | X \right)=\frac{1}{1+exp(-\alpha_{0}-\alpha_{1}X_{1}-\ldots-\alpha_{n}X_{n})}$$

In this formula, $\alpha_{0}$ represents the intercept, and $\alpha_{j} (1 \leq j \leq n)$ denotes the weight coefficient for each category of the feature. $X_{j} (1 \leq j \leq n)$ corresponds to each category of the features, and $Y$ is a binary outcome. The weight coefficient determines the score for each category. After fitting the data to the multivariable logistic regression model, the coefficient of $\alpha_{j}$ is obtained. The category with the lowest $\alpha_{j}$ is set as the reference category (e.g., the coefficient of the reference is set as 0). The logistic regression is then rerun with this adjusted reference to ensure no negative coefficients. After that, each $\alpha_{j}$ is divided by the lowest coefficient $\alpha_{low}$. The weighted points for each category are defined as $\alpha_{j}\left( score \right)=round(\frac{\alpha_{j}}{\alpha_{low}})$. This results in a scoring table where each category is assigned points based on its coefficient. The total score is computed by summing all points to meet specific clinical application needs.

**Module 4: Model Selection and Parameter Determination**

The model is optimized to achieve a balance between simplicity and high predictive accuracy. This is accomplished by testing different feature counts in the training set and visualizing the relationship between model performance and complexity using a parsimony plot. The optimal number of features is identified at the point when adding additional features no longer yields a significant improvement in performance. To finalize the feature set, features are incrementally added and evaluated based on their contribution to increasing the area under the receiver of characteristic curve (AUC-ROC). If these additions necessitate adjustments, Modules 2 and 3 are re-executed to refine the model. After confirming the optimal feature set, the final list of features is determined based on their rankings from Module 1.

**Module 5: Predictive Performance Evaluation**

The performance of the developed model is evaluated using the test set, with the AUC-ROC serving as the primary evaluation metric. In addition, sensitivity, specificity, positive predictive value (PPV), and negative predictive value (NPV) are calculated to provide a comprehensive assessment of the model's performance. Where applicable, external validation is conducted using an independent dataset to further evaluate the model's robustness and generalizability.

**eMethods - The A4-Study description**

The A4-Study is a multicenter clinical trial conducted across 67 sites in the United States, Canada, Australia, and Japan, enrolling 1,169 participants aged 65 to 85 years by 2017. The trial aims to slow cognitive decline in individuals at the preclinical stage of Alzheimer’s disease—those who were cognitively normal, living independently, and accompanied by a study partner for annual cognitive assessments, but with elevated amyloid levels confirmed through PET imaging. Eligibility criteria included screening for Logical Memory Delayed Recall (LMDR-IIa) scores to exclude individuals with very low scores (≤1.5 SD below norms), indicative of mild cognitive impairment (MCI), and very high scores (>1.5 SD above norms), which were excluded to enrich the cohort for individuals more likely to have elevated amyloid and to avoid ceiling effects associated with unusually preserved memory performance [6, 7]. Additional requirements included a Clinical Dementia Rating (CDR) score of 0, a Mini-Mental State Examination (MMSE) score between 25 and 30, and LMDR-IIa scores between 6 and 18. At baseline, all participants were assessed using PACC, CDR, C3, Florbetapir PET, tau PET, pTau-217, and MRI, with periodic reassessments conducted throughout the placebo-controlled period (week 1 to week 240) and a final assessment at week 240.

In this study, all the participants have full records of required features, and no imputation has been used in the data processing step.

**eTable 1: Initial features included in this study.**

The initial features included in this study can be available in the excel sheet at: [eTable 1.csv](https://1drv.ms/x/c/57df6b337432cded/EdaFDgvi2Y9LgoUX0TJt5PEB09iQQLM-0Yq4-GEeJtf_3A?e=Ggg3aH).

**eTable 2: Score table for modified ASAB models.**

| **(A) Model without baseline brain amyloid burden (threshold 63)** | | |
| --- | --- | --- |
| **Baseline feature** | **Interval** | **Score** |
| pTau-217 level (U/mL) | < 0.2 | 0 |
|  | [0.2, 0.25) | 20 |
|  | [0.25, 0.34) | 37 |
|  | ≥ 0.34 | 32 |
| Blood test – ALT (U/L) | < 15 | 7 |
|  | [15, 18) | 10 |
|  | [18, 22) | 12 |
|  | ≥ 22 | 0 |
| Blood test - creatine kinase (U/L) | < 73 | 0 |
|  | [73, 98) | 13 |
|  | [98, 135) | 18 |
|  | ≥ 135 | 4 |
| Blood test – platelets (10^9^/L) | < 190 | 0 |
|  | [190, 223) | 7 |
|  | [223, 266) | 14 |
|  | ≥ 266 | 18 |
| PACC score | < -1.59 | 5 |
|  | [-1.59, 0.21) | 0 |
|  | [0.21, 1.94) | 0 |
|  | ≥ 1.94 | 5 |
| Blood test – cholesterol (mmol/L) | < 4.37 | 0 |
|  | [4.37, 4.97) | 2 |
|  | [4.97, 5.54) | 4 |
|  | ≥ 5.54 | 10 |
| **(B) Model without baseline plasma pTau-217 (threshold 53)** | | |
| **Baseline feature** | **Interval** | **Score** |
| Amyloid beta SUVR | < 1.21 | 0 |
|  | [1.21, 1.32) | 23 |
|  | [1.32, 1.45) | 50 |
|  | ≥ 1.45 | 68 |
| Blood test – calcium (U/L) | < 2.3 | 6 |
|  | [2.30, 2.37) | 1 |
|  | [2.37, 2.42) | 13 |
|  | ≥ 2.42 | 0 |
| Blood test - MCV (f/L) | < 88 | 14 |
|  | [88, 91) | 5 |
|  | [91, 93.5) | 5 |
|  | ≥ 93.5 | 0 |
| AST/ALT ratio | < 1.04 | 1 |
|  | [1.04, 1.25) | 0 |
|  | [1.25, 1.44) | 5 |
|  | ≥ 1.44 | 3 |

Abbreviations: ALT **=** alanine aminotransferase; AST = aspartate aminotransferase; MCV **=** mean corpuscular volume; PACC = preclinical Alzheimer cognitive composite

**eTable 3: Score tables for modified ASPT models.**

| 1. **Model without baseline brain amyloid burden (threshold 51)** | | |
| --- | --- | --- |
| **Baseline feature** | **Interval** | **Score** |
| pTau-217 level (U/mL) | < 0.193 | 0 |
|  | [0.193, 0.242) | 32 |
|  | [0.242, 0.338) | 45 |
|  | ≥ 0.338 | 60 |
| Blood test - creatine kinase (U/L) | < 70 | 12 |
|  | [70, 95) | 5 |
|  | [95, 135) | 8 |
|  | ≥ 135 | 0 |
| Blood test – eosinophils (10^9^/L) | < 0.09 | 0 |
|  | [0.09, 0.14) | 15 |
|  | [0.14, 0.21) | 10 |
|  | ≥ 0.21 | 8 |
| Blood test – phosphorus (mmol/L) | < 1.03 | 0 |
|  | [1.03, 1.13) | 2 |
|  | [1.13, 1.23) | 5 |
|  | ≥ 1.23 | 5 |
| Blood test – GGT (U/L) | < 13 | 5 |
|  | [13, 18) | 0 |
|  | [18, 25) | 8 |
|  | ≥ 15 | 5 |
| **(B) Model without baseline plasma pTau-217 levels (threshold 46)** | | |
| **Baseline feature** | **Interval** | **Score** |
| Amyloid beta (SUVR) | < 1.21 | 0 |
|  | [1.21, 1.33) | 24 |
|  | [1.33, 1.46) | 32 |
|  | ≥ 1.46 | 38 |
| Blood test - WBC (10^9^/L) | < 4.91 | 27 |
|  | [4.91, 5.87) | 16 |
|  | [5.87, 6.98) | 0 |
|  | ≥ 6.98 | 11 |
| Blood test - creatine kinase (U/L) | < 69 | 22 |
|  | [69, 93) | 16 |
|  | [93, 130) | 14 |
|  | ≥ 130 | 0 |
| Age (year) | < 68 | 3 |
|  | [68, 71.6) | 0 |
|  | [71.6, 75.1) | 3 |
|  | ≥ 75.1 | 14 |

Abbreviations: GGT **=** Gamma-glutamyl transferase, WBC **=** White Blood Cell

**eTable 4: Epidemiology analysis.**

| **(A) Association of baseline features with brain amyloid burden (SUVR) after 4.5 years** | |
| --- | --- |
|  | **β (SE), [95%CI], *P*-value** |
| Baseline amyloid beta SUVR | 0.808 (0.029), [0.751, 0.864], *P*<.001 |
| Baseline pTau-217 level | 0.331 (0.047), [0.239, 0.423], *P*<.001 |
| PACC score | -0.171 (0.052), [-0.272, -0.069], *P=*.001 |
| Blood test-platelets | 0.018 (0.052), [-0.085, 0.120], *P*=.73 |
| Blood test - ALT | -0.136 (0.049), [-0.232, -0.039], *P*=.006 |
| Blood test - cholesterol | 0.001 (0.052), [-0.102, 0.104], *P*=.98 |
| Blood test - creatine kinase | -0.054 (0.050), [-0.152, 0.044], *P*=.28 |
| **(B) Association of baseline features with plasma pTau-217 levels after 4.5 years** | |
|  | **β (SE), [95%CI], *P*-value** |
| Baseline amyloid beta SUVR | 0.432 (0.054), [0.326, 0.538], *P*<.001 |
| Baseline pTau-217 level | 0.837 (0.044), [0.751, 0.923], *P*<.001 |
| PACC score | -0.191 (0.060), [-0.310, -0.072], *P*=.002 |
| Blood test - cholesterol | -0.082 (0.059), [-0.198, 0.033], *P*=.16 |
| Blood test - creatine kinase | -0.105 (0.056), [-0.216, 0.005], *P*=.06 |
| R-R interval | 0.112 (0.059), [-0.005, 0.228], *P*=.06 |
| Blood test - eosinophils | -0.020 (0.059), [-0.136, 0.097], *P*=.74 |
| Blood test - phosphorus | 0.066 (0.063), [-0.059, 0.190], *P*=.30 |
| Blood test - GGT | -0.091 (0.057), [-0.204, 0.021], *P*=.11 |

Note: All associations were adjusted for age, sex, education, and APOE. Significant associations were marked in red.

**
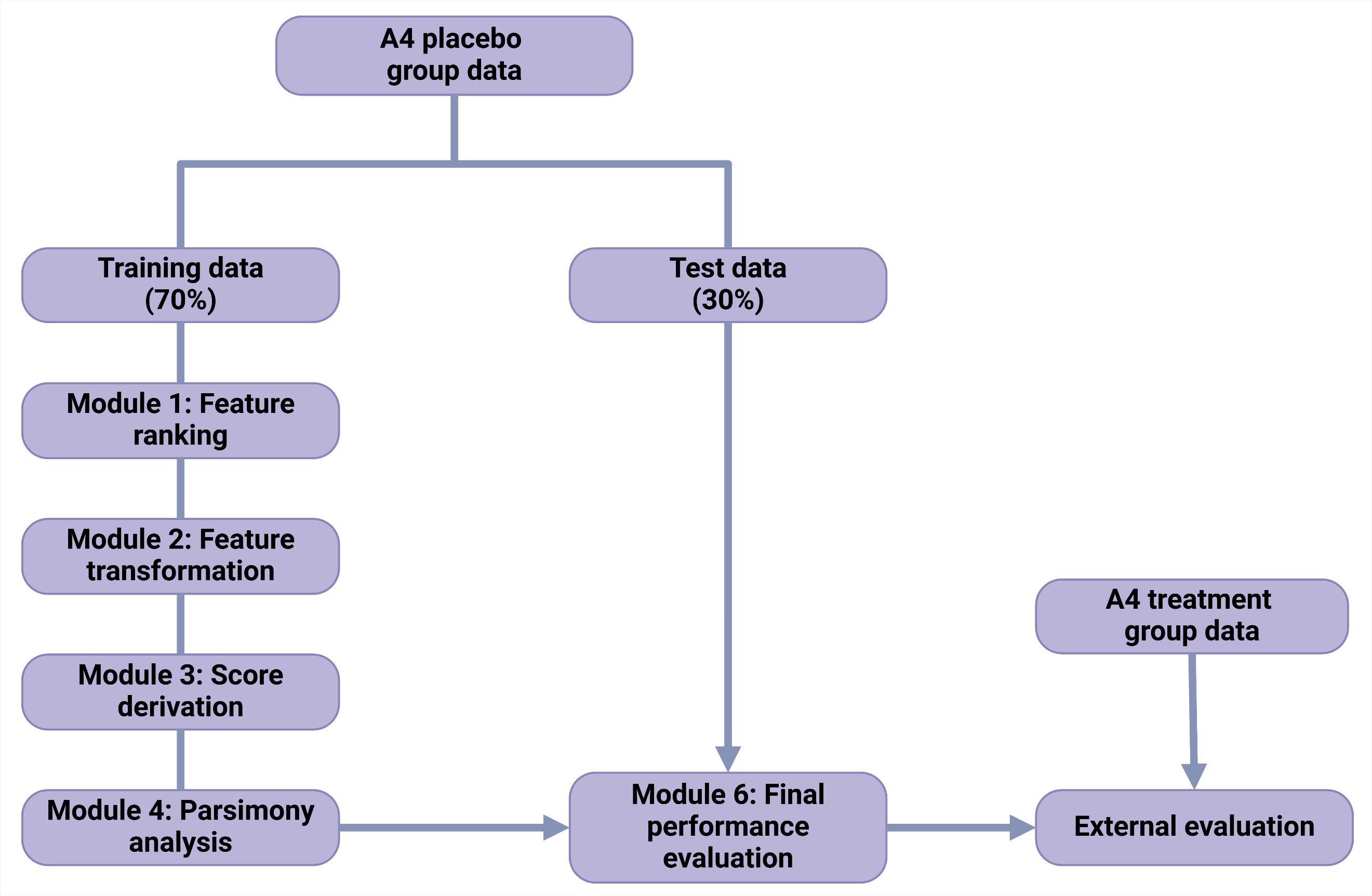
**

**eFigure 1: Flowchart of the model development.**

**
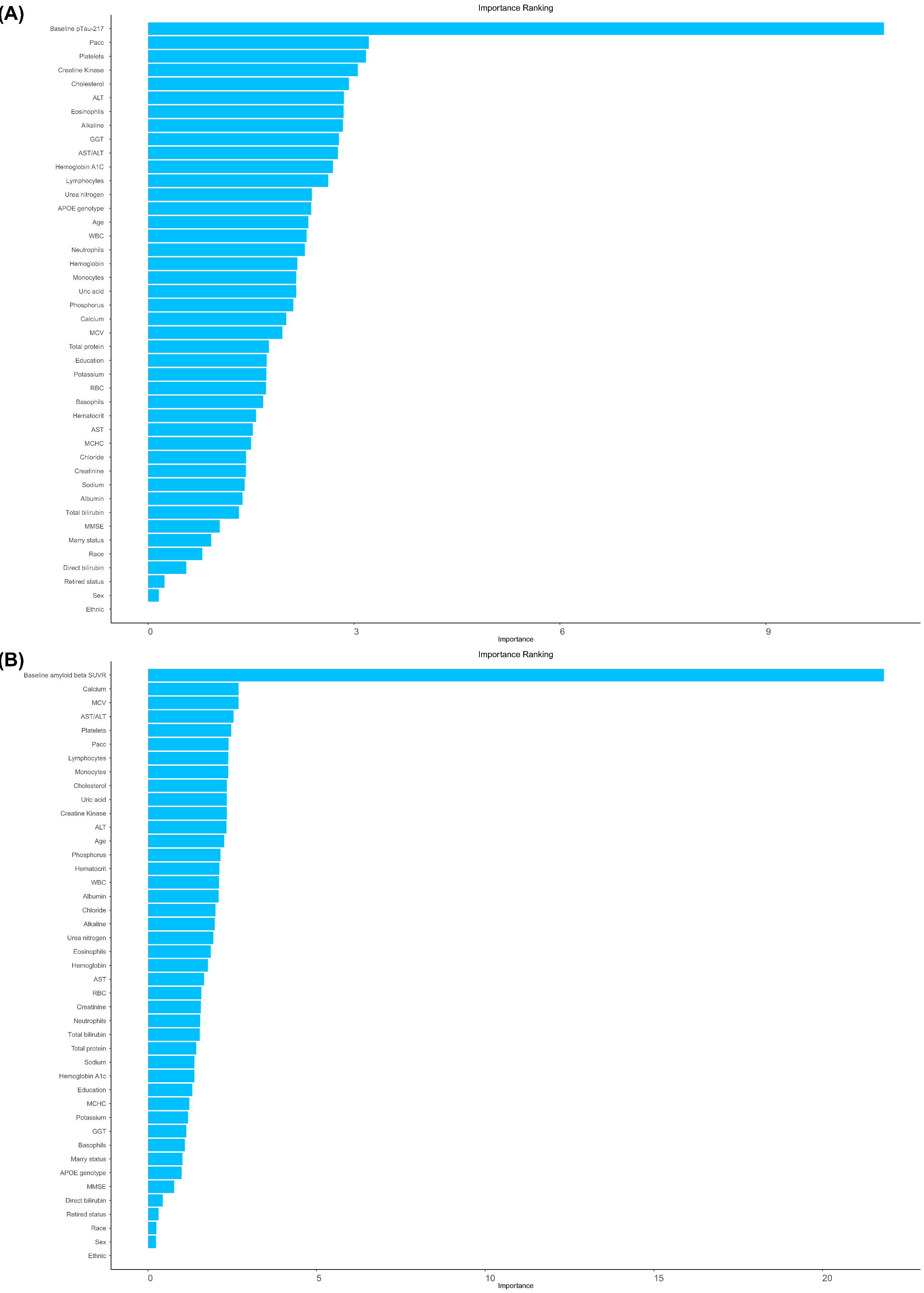
**

**eFigure 2: Feature ranking for modified ASAB models.** (A) Feature importance ranking for the ASAB model excluding baseline amyloid-beta SUVR. (B) Feature importance ranking for the ASAB model excluding baseline pTau-217 levels.

**
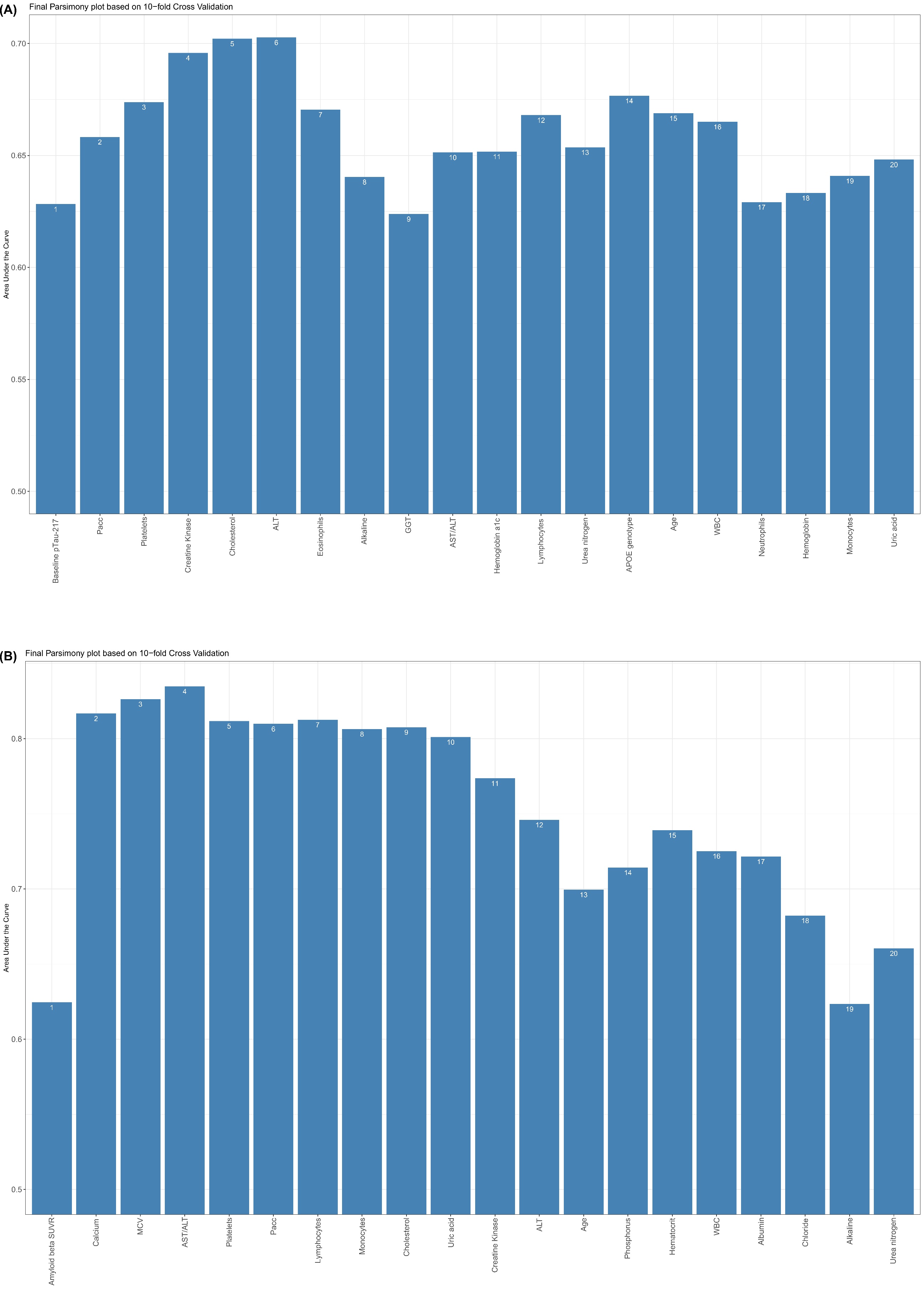
**

**eFigure 3: Parsimony plots for modified ASAB models.** (A) Parsimony plot for the ASAB model excluding baseline amyloid-beta SUVR. (B) Parsimony plot for the ASAB model excluding baseline pTau-217 levels.

**
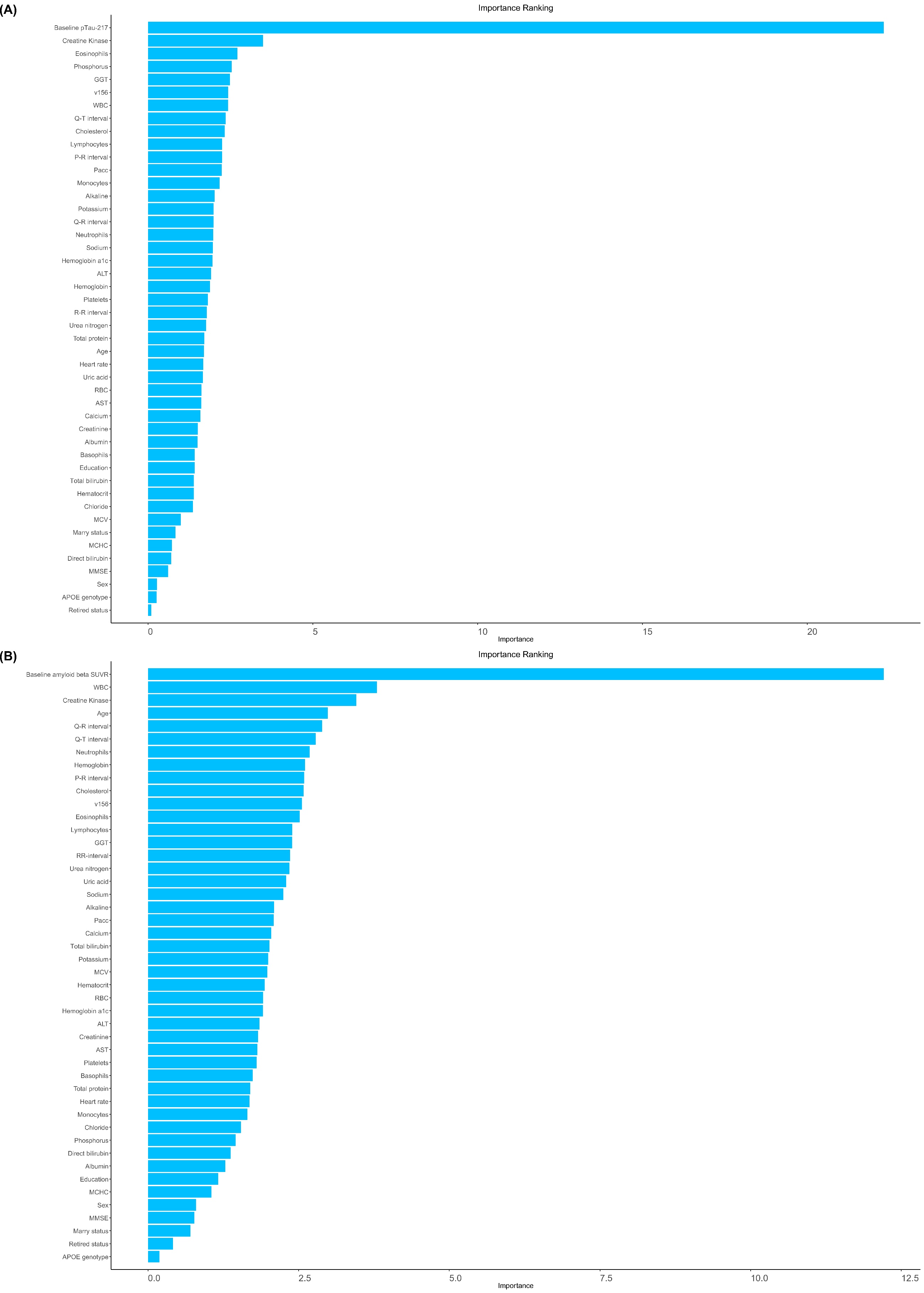
**

**eFigure 4: Feature ranking for modified ASPT models.** (A) Feature importance ranking for the ASPT model excluding baseline amyloid-beta SUVR. (B) Feature importance ranking for the ASPT model excluding baseline pTau-217 levels.

**
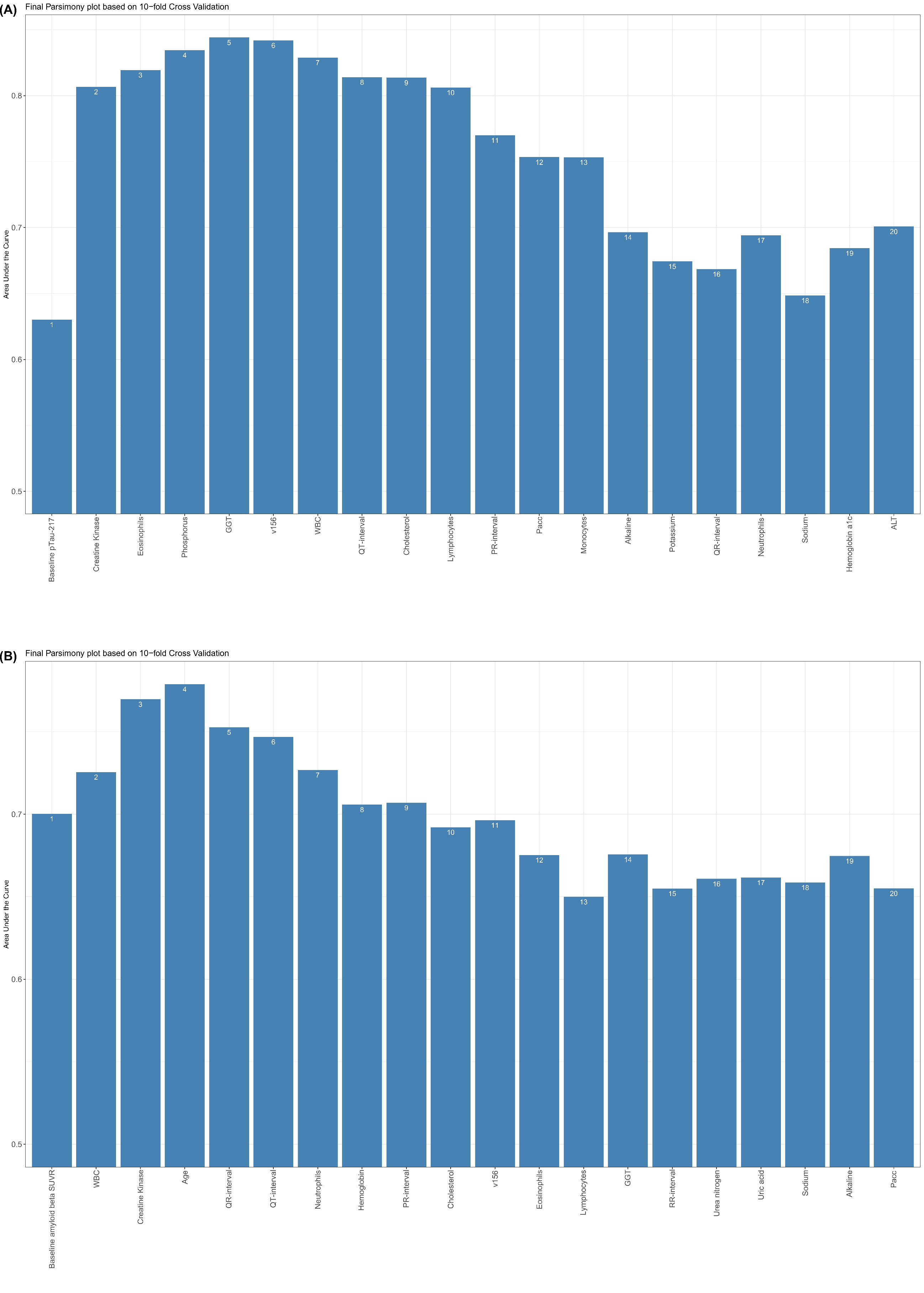
**

**eFigure 5: Parsimony plots for modified ASPT models.** (A) Parsimony plot for the ASPT model excluding baseline amyloid-beta SUVR. (B) Parsimony plot for the ASPT model excluding baseline pTau-217 levels.

Reference

1. Xie F, Ning Y, Liu M, Li S, Saffari SE, Yuan H, Volovici V, Ting DSW, Goldstein BA, Ong MEH: A universal AutoScore framework to develop interpretable scoring systems for predicting common types of clinical outcomes. *STAR Protocols* 2023, 4(2):102302.

2. Pretorius A: Advances in random forests with application to classification. Stellenbosch: Stellenbosch University; 2016.

3. Kayri M, Kayri İ: The comparison of Gini and Twoing algorithms in terms of predictive ability and misclassification cost in data mining: an empirical study. *Databases* 2015, 3:5.

4. Sorochan Armstrong MD, de la Mata AP, Harynuk JJ: Review of variable selection methods for discriminant-type problems in chemometrics. *Frontiers in Analytical Science* 2022, 2:867938.

5. Bouwmeester W, Zuithoff NP, Mallett S, Geerlings MI, Vergouwe Y, Steyerberg EW, Altman DG, Moons KG: Reporting and methods in clinical prediction research: a systematic review. *PLoS Medicine* 2012, 9(5):e1001221.

6. Sperling RA, Donohue MC, Raman R, Rafii MS, Johnson K, Masters CL, van Dyck CH, Iwatsubo T, Marshall GA, Yaari R: Trial of solanezumab in preclinical Alzheimer’s disease. *New England Journal of Medicine* 2023, 389(12):1096-1107.

7. Grober E, Lipton RB, Sperling RA, Papp KV, Johnson KA, Rentz DM, Veroff AE, Aisen PS, Ezzati A: Associations of stages of objective memory impairment with amyloid PET and structural MRI: The A4 study. *Neurology* 2022, 98(13):e1327-e1336.
